# Evaluation of the flipped dose NIVO3+IPI1 in patients with advanced unresectable melanoma

**DOI:** 10.1093/jnci/djaf327

**Published:** 2025-12-08

**Authors:** Karl Björkström, Cissi Liu, Anna Fager, Lisa L Liu, Lars Ny, Hildur Helgadottir

**Affiliations:** Department of Oncology-Pathology, Karolinska Institutet, Stockholm, Sweden; Theme Cancer, Karolinska University Hospital, Stockholm, Sweden; Department of Oncology-Pathology, Karolinska Institutet, Stockholm, Sweden; Department of Oncology, Institute of Clinical Sciences, Sahlgrenska Academy, University of Gothenburg, Gothenburg, Sweden; Department of Oncology, Sahlgrenska University Hospital, Gothenburg, Sweden; Department of Oncology-Pathology, Karolinska Institutet, Stockholm, Sweden; Theme Cancer, Karolinska University Hospital, Stockholm, Sweden; Department of Clinical Science, Intervention and Technology, Karolinska Institutet, Stockholm, Sweden; Department of Oncology, Institute of Clinical Sciences, Sahlgrenska Academy, University of Gothenburg, Gothenburg, Sweden; Department of Oncology, Sahlgrenska University Hospital, Gothenburg, Sweden; Department of Oncology-Pathology, Karolinska Institutet, Stockholm, Sweden; Theme Cancer, Karolinska University Hospital, Stockholm, Sweden

## Abstract

Nivolumab 1 mg/kg plus ipilimumab 3 mg/kg (NIVO1+IPI3) was approved for advanced melanoma in 2016. The CheckMate 511 trial demonstrated improved tolerability with the flipped dose, NIVO3+IPI1, but this regimen has not been approved in melanoma by regulatory authorities. In this study, patients with advanced unresectable melanoma treated with NIVO3+IPI1 or NIVO1+IPI3 were included. The objective response rate was 48.8% with NIVO3+IPI1 (*n* = 209) and 36.9% with NIVO1+IPI3 (*n* = 190) (*P *= .016). Adjusted hazard ratio (aHR) was 0.67 (95% CI = 0.53 to 0.87, *P *= .002) for progression-free survival and 0.59 (95% CI = 0.44 to 0.78, *P *< .001) for overall survival (OS). In most studied subgroups aHR was <1, in favor of NIVO3+IPI1. The incidence of grade 3-5 immune-related adverse events was 30.6% with NIVO3+IPI1 vs. 51.1% with NIVO1+IPI3 (*P < *.001). This study shows that in a real-world setting, NIVO3+IPI1 demonstrated superior efficacy compared with NIVO1+IPI3, possibly related to a beneficial safety and tolerability profile allowing for more received doses.

The combination of the PD-1 and the CTLA-4 immune checkpoint inhibitors (ICI) nivolumab and ipilimumab has emerged as the most effective treatment in patients with advanced melanoma, demonstrating melanoma-specific survival of 52% at 10 years in the pivotal CheckMate 067 trial.^2^ However, the efficacy of the nivolumab 1 mg/kg and ipilimumab 3 mg/kg combination (NIVO1+IPI3) comes with significant toxicity; 62% experienced grade 3-4 immune-related adverse events (irAEs), and 34% discontinued treatment due to these irAEs.^1^ To address the high incidence of severe irAEs, Lebbe et al conducted the CheckMate 511 trial to evaluate an alternative “flipped” dosing regimen in patients with unresectable metastatic melanoma: nivolumab 3 mg/kg and ipilimumab 1 mg/kg (NIVO3+IPI1).^2^ This dose had previously been used in renal cell carcinoma with a substantially lower frequency of irAEs, and is the dosing that has been approved in other cancers, including hepatocellular carcinoma and lung cancer.^3^^,^^4^ In the CheckMate 511 study, the overall response rate (ORR) was 46% with the NIVO3+IPI1 dose and 51% with the NIVO1+IPI3 dose. Further, 2 studies evaluated ipilimumab 1 mg/kg in combination with a PD-1 inhibitor as second line treatment after progression on PD-1 inhibitors, and found an ORR of 28% (nivolumab plus ipilimumab, SWOG S1616) and 29% (pembrolizumab plus ipilimumab).^5^^,^^6^ These studies demonstrated that such dosing can also be effective in PD-1 refractory melanoma, and the SWOG S1616 study also showed a superior outcome compared with monotherapy with ipilimumab 3 mg/kg, where ORR was only 9%. Moreover, for neoadjuvant treatment of resectable melanoma, the NIVO3+IPI1 regimen in the OpACIN-neo trial was found to give similar rates of pathological response but a significantly lower rate of irAEs, compared with NIVO1+IPI3.^7^ The NIVO3+IPI1 dose was therefore chosen as the neoadjuvant regimen for the NADINA study,^8^ which has recently been implemented in the neoadjuvant melanoma treatment in some countries, including the Netherlands, Sweden, and Australia.

The CheckMate 511 study also demonstrated a significantly lower rate of grade 3-5 irAEs in patients receiving the flipped dose compared with the traditional dose (34% vs. 48%). Although no significant survival differences were seen at a median follow-up of 19 months, the CheckMate 511 trial that included 360 patients was not designed to demonstrate non-inferiority for efficacy, and the NIVO3+IPI1 regimen has not been approved for advanced melanoma by regulatory authorities. Further, in the KEYNOTE-029 trial, standard-dose pembrolizumab plus 4 doses of either 50 mg or 100 mg ipilimumab was evaluated in 51 + 51 patients with metastatic melanoma, demonstrating a higher rate of grade 3–5 irAEs events with 100 mg ipilimumab, but similar anti-tumoral efficacy.^9^

After the reporting of the CheckMate 511 study in 2019, the NIVO3+IPI1 regimen has been used in some clinics and in some countries, but to our knowledge, there have been no reports comparing the NIVO3+IPI1 to the NIVO1+IPI3 dosing regimen in a real-world setting. NIVO3+IPI1 was introduced in 2019 as an alternative dosing regimen in Sweden, approved by the national treatment guidelines. Due to concerns regarding the high rate and severity of side effects experienced with the NIVO1+IPI3 regimen, NIVO3-IPI1 has been widely used in patients with advanced melanoma, also those with brain metastases, and not reserved for, for example, frailer patients where there are concerns regarding treatment tolerability. We here report clinical outcomes of patients with advanced unresectable melanoma treated with either traditional or the flipped dose of NIVO+IPI (see the Supplementary Materials for details on patients and methods).

We analyzed 399 patients with advanced unresectable non-uveal melanoma, 209 receiving NIVO3+IPI1 (median follow-up 41 months, IQR 20-86 months) and 190 receiving NIVO1+IPI3 (median follow-up 53 months, IQR 19-130 months). Baseline characteristics of both treatment groups are presented in Table S1. Although there were no significant differences in sex, ECOG performance status, baseline LDH level, BRAF mutation status, or having previous treatments, the median age was higher in the NIVO3+IPI1 group compared with the NIVO1+IPI3 group (63 vs 60 years, *P < *.001). The proportion with M1c disease was similar (36.8% vs 32.1%), whereas the NIVO3+IPI1 group had a higher proportion of patients with earlier-stage disease (stage III, M1a and M1b: 42.1% vs 17.9%) and the NIVO1+IPI3 group had a significantly higher proportion of patients with brain metastases (M1d: 50% vs 21.1%) (*P < *.001). Looking at the specific sites of the metastases, there were no significant differences in having bone, liver or other visceral metastases, while the NIVO3+IPI1 patients had more soft tissue metastases (*P *= .027) and the NIVO1+IPI3 patients had more lung (*P *= .035) and CNS (*P *> .001) metastases. The NIVO1+IPI3 patients had a median of 3 (IQR 2-4) and the NIVO3+IPI1 had a median of 2 (IQR 1-3) metastatic sites (*P* < .001). Among the patients with brain metastases (*n* = 139), there was no statistical difference between the 2 treatment cohorts regarding numbers or size of CNS metastases, whether they were symptomatic or whether they had been treated with radical surgery or stereotactic radiotherapy or radiosurgery before the treatment started (Table S2). Overall response rate was significantly higher in the NIVO3+IPI1 group compared with the NIVO1+IPI3 group (48.8% [95% CI = 43.2 to 56.8] vs. 36.9% [95% CI = 31.1 to 45.1], *P* = .016) (Table S2). Disease control rate was also significantly higher in the NIVO3+IPI1 group (60.3% [95% CI = 54.9 to 68.2] vs. 45.8% [95% CI = 39.9 to 54.3], *P = *.004). Complete responses were observed in 23.0% of patients in the NIVO3+IPI1 group compared with 15.3% in the NIVO1+IPI3 group.

Median OS was also significantly longer in the NIVO3+IPI1 group compared with the NIVO1+IPI3 group (42.4 months [95% CI = 27.8 to NR] vs. 14.5 months [95% CI = 8.5 to 19.8]), *P = *.003 (see Kaplan-Meier plot in Figure S1).

After adjusting for baseline age, LDH levels, ECOG, disease stage, and numbers of metastatic sites, the NIVO3+IPI1 regimen was still associated with a significantly reduced risk of death compared with the NIVO1+IPI3 regimen (adjusted hazard ratio [aHR] 0.59, 95% CI = 0.44 to 0.78, *P *< .001) (Figure 1A). The median PFS was likewise significantly longer in the NIVO3+IPI1 group compared with the NIVO1+IPI3 group (8.9 months [95% CI = 5.8 to 14.8] vs 2.7 months [95% CI = 2.2 to 4.8] *P* = .011) (Figure S1). After adjustment for the same baseline characteristics, the NIVO3+IPI1 regimen was still associated with a significantly reduced risk of disease progression (aHR 0.67, 95% CI = 0.53 to 0.87, *P* = .002) (Figure 1B). Subgroup analyses of OS and PFS were conducted to evaluate treatment effects across different patient populations (see forest plots in Figure 1 and Kaplan-Meier graphs in Figures S2 and S3). The pattern, in favor of the NIVO3+IPI1 regimen, was consistent across most subgroups.

**Figure 1.**
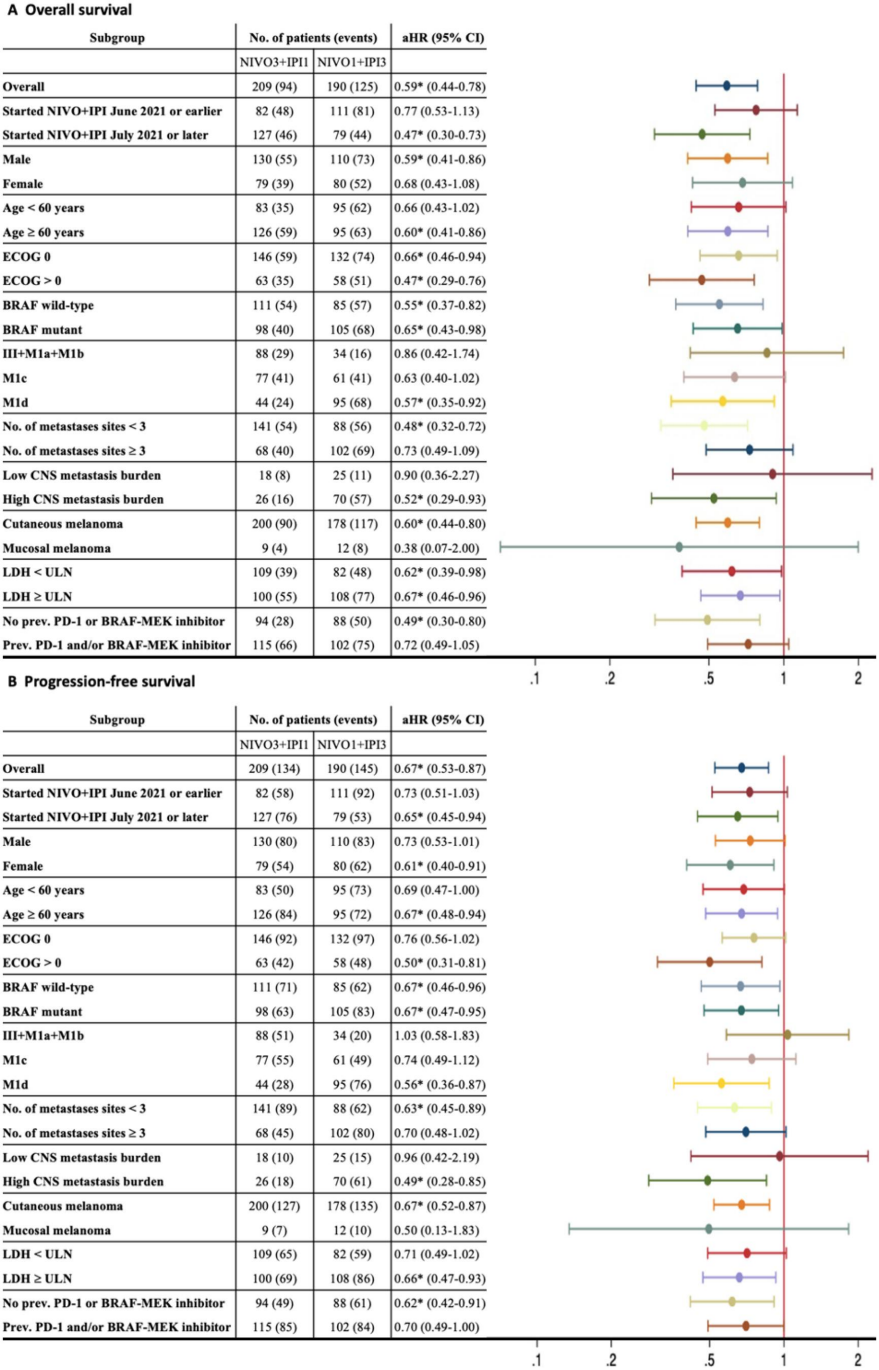
Forest plot showing adjusted hazard ratios for **(A)** overall survival and **(B)** progression-free survival with 95% confidence intervals comparing NIVO3+IPI1 versus NIVO1+IPI3 across subgroups of patients with advanced melanoma. *P-values < .05. *Low CNS metastases burden: patients with all CNS metastases treated with surgery or stereotactic radiotherapy/stereotactic radiosurgery (SRT/SRS) before start of NIVO+IPI treatment OR those with a maximum of 2 CNS metastases, each less than 1 cm AND not symptomatic (patients not on cortisone). High CNS burden: all patients not falling into the criteria for low CNS burden.

The incidence of grade 3-5 irAEs was significantly lower in the NIVO3+IPI1 group compared with the NIVO1+IPI3 group (30.6% vs. 51.1%, *P < *.001) (Table S3). In the NIVO3+IPI1- and NIVO1+IPI3-treated patients, 32.1% and 36.8% discontinued treatment due to irAEs, where 16.7% and 27.3% discontinued because of grade 3–5 irAEs in the 2 groups, respectively. Discontinuation because of irAEs was after a median of 3 doses with NIVO3+IPI1, and after 2 doses with NIVO1+IPI3. Analysis of treatment exposure (Figure 2) revealed that patients in the NIVO3+IPI1 group completed all 4 doses of combination therapy significantly more frequently than those in the NIVO1+IPI3 group, 57.4% vs 33.7% (*P *= .005). Furthermore, a significantly higher proportion of patients in the NIVO3+IPI1 group received maintenance nivolumab therapy: 45.5% vs 27.4% (*P* = .01) received at least 3 doses. As for post-progression treatments, 39.2% and 47.4% had later treatments in the NIVO3+IPI1 and NIVO1+IPI3 groups, respectively (*P *= .104). In the 2 groups, 23.9% and 24.7% had BRAF (±MEK) inhibitors, 4.8% and 4.7% had rechallenge with PD-1 inhibitor monotherapy, 3.3% and 5.3% had rechallenge with PD-1 and CTLA-4 inhibitor combination therapy, and 11.5% and 24.2% had chemotherapy, respectively (Table S4).

**Figure 2.**
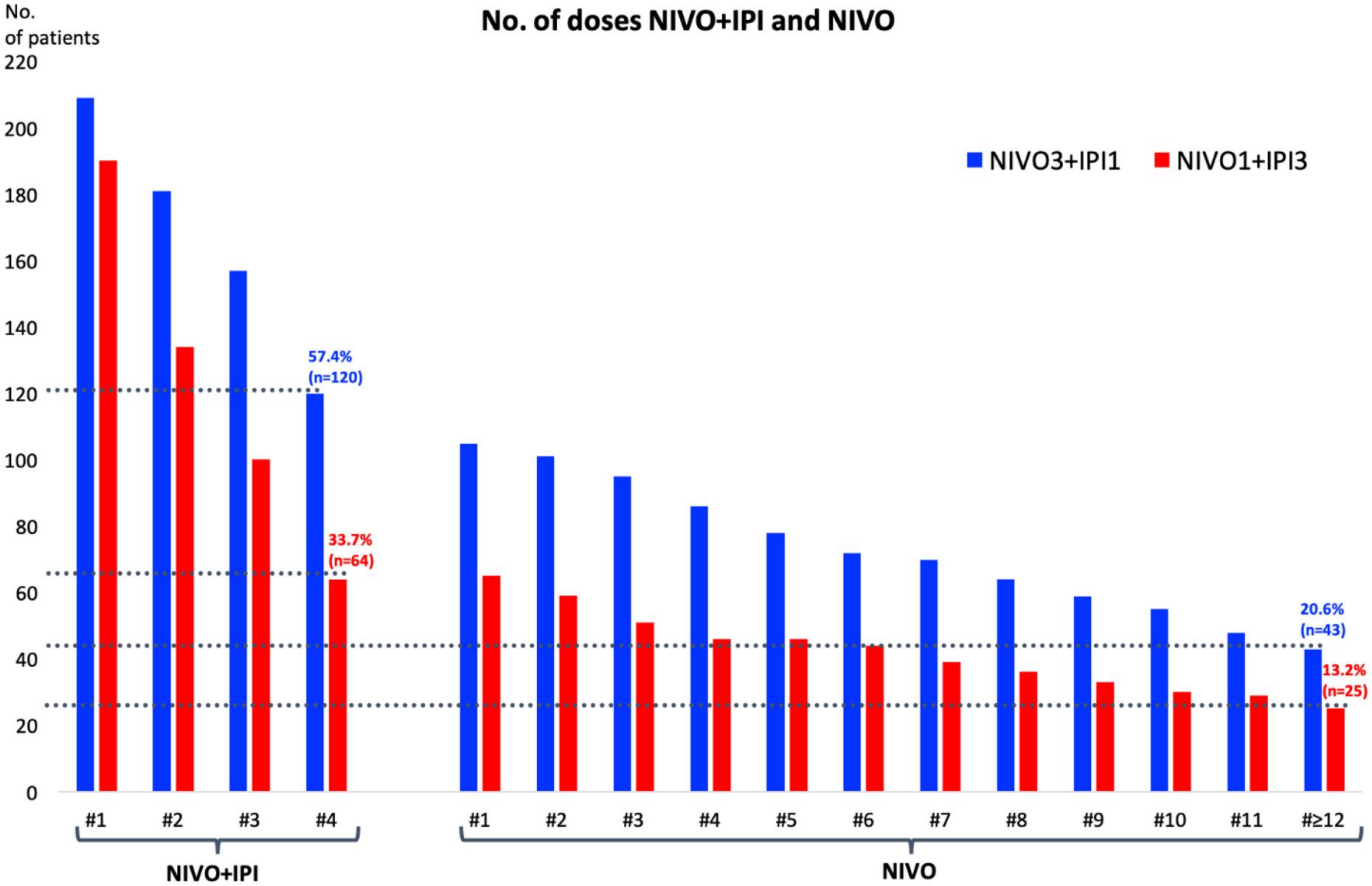
Bar chart showing the number of patients receiving each dose of combination therapy (NIVO+IPI, doses #1-4) and subsequent maintenance nivolumab monotherapy (NIVO, doses #1-≥12) in the NIVO3+IPI1 and NIVO1+IPI3 groups. Number with percentage indicates the proportion of patients who completed all combination doses or received extended maintenance therapy.

Besides the CheckMate 511 trial (where no follow-up has been reported beyond the initial 19 months), there have been no other prospective or retrospective studies comparing NIVO3+IPI1 and NIVO1+IPI3 in advanced melanoma. In contrast to earlier assumptions, this real-world report of patients with advanced melanoma showed that NIVO3+IPI1 achieved superior efficacy compared with NIVO1+IPI3. Also, after adjustments for baseline factors and in subgroup analyses, the significant advantage of the NIVO3+IPI1 regimen remained. In subgroups where other studies have indicated a particular advantage of NIVO1+IPI3 compared with nivolumab monotherapy, including those with brain metastases, BRAF mutated tumors, elevated LDH and mucosal melanoma, there was a significant advantage in favor of the NIVO3+IPI1 in our cohort.^1^^,^^10^ Importantly, NIVO3+IPI1 was associated with substantially fewer grade 3–5 adverse events, enabling significantly higher treatment completion rates. The longer treatment exposure, we think, is the most plausible explanation for the better treatment outcome with the NIVO3+IPI1 regimen in a real-world setting. Yet, the effect of treatment exposure over time is still not well studied. The DANTE trial, however, demonstrated a less favorable outcome in melanoma patients stopping on PD-1 inhibitors after 1 year compared with continuing beyond 1 year (HR = 3.0; 90% CI = 1.0 to 8.5). However, this was an underpowered study (*n* = 83 + 83) that did not reach its inclusion goals due to recruitment difficulties.^11^ Further, a retrospective trial assessing melanoma patients treated with NIVO+IPI without tumor progression that developed irAEs requiring treatment interruption reported a significantly better OS in those that resumed treatment with nivolumab monotherapy compared with those that did not resume.^12^

The population-based cohort, including patients with brain metastases, prior treatments and poor performance status, typically excluded from clinical trials, gives insights into the treatment efficacy and tolerability with these drugs in routine clinical practice. The NIVO3+IPI1 has not been extensively studied in patients with brain metastases; however, a study including 19 ICI naïve melanoma patients with brain metastases receiving low dose ipilimumab in combination with pembrolizumab found an intracranial response rate of 43%.^13^ These data are in line with this study, where results on flipped dosing also demonstrate clinically meaningful efficacy that was even superior compared with traditional dosing in patients with high CNS metastases burden.

It is, however, important to acknowledge that this study has limitations. The retrospective design limits our ability to establish causality, and the treatment groups differed significantly in baseline characteristics, particularly disease stage distribution as well as the timing of inclusion. The efficacy differences of the 2 regimes could theoretically have been due to increased clinical experience with ICI over time among the healthcare personnel, including improved management of irAEs, potentially leading to better outcomes with the NIVO3+IPI1 that was implemented later. Still, the timing of the treatment revealed similar patterns, with HR < 1 for both OS and PFS, in favor of NIVO1+IPI3 (Figure 1). Further, it was somewhat unexpected that while the Checkmate 511 trial demonstrated numerically similar efficacy with the 2 doses, our study showed a benefit of the NIVO3+IPI1 dose. This could be related to the real-world setting in our study with, for example, worse irAE tolerance in such a population, and although we adjusted for multiple factors, we cannot preclude that this effect results from unmeasurable confounding, and that, for example, physicians may have chosen NIVO1+IPI3 for patients they perceive to have more aggressive disease. Further, the study only included patients from one country, with implications on the generalizability.

We believe that the findings nonetheless have clinical relevance as they raise concerns regarding possible overtreatment with the higher dose of ipilimumab in the traditional NIVO1+IPI3 regimen in advanced melanoma, and provide evidence supporting the use of NIVO3+IPI1 as a preferred regimen that may offer both improved efficacy and tolerability. Continued evaluation of the flipped dose NIVO3+IPI1 regimen in advanced melanoma is warranted, as well as its efficacy and tolerability compared with the recently approved nivolumab and LAG3 inhibitor relatlimab combination.^14^

## Supplementary Material

djaf327_Supplementary_Data

## Data Availability

The data that support the findings of this study are available from the corresponding author upon reasonable request.
