# Supplementary material for: Evaluation of the flipped dose NIVO3+IPI1 in patients with advanced unresectable melanoma
Source: J Natl Cancer Inst. 2025 Dec 8;118(7):1320–4. doi: 10.1093/jnci/djaf327 (PMC13339152; doi:10.1093/jnci/djaf327)
Supplement: djaf327_Supplementary_Data [file djaf327_supplementary_data.zip › djaf327_Supplementary_Data.docx]

Karl Björkström, Cissi Liu, Anna Fager, Lisa L Liu, Lars Ny, Hildur Helgadottir

**Evaluation of the flipped dose NIVO3+IPI1 in patients with advanced unresectable melanoma**

**Supplementary file:**

**Supplementary Methods**

**Table S1: Baseline characteristics**

**Table S2: Treatment Response**

**Table S3: Adverse events**

**Table S4. Post-progression treatment**

**Figure S1: Kaplan-Meier: All and M1d excluded**

**Figure S2: Kaplan-Meier: BRAF mut and wt**

**Figure S3: Kaplan-Meier: Different stages**

**Supplementary Methods**

*Participants and study design*

This is a retrospective cohort study including all patients diagnosed with advanced melanoma treated with NIVO+IPI at Karolinska University Hospital, Stockholm, Sweden and Sahlgrenska University Hospital, Gothenburg, Sweden. The melanoma oncology is centralized to these clinics, and the study is hence a population-based study from the Stockholm and Gothenburg regions with altogether nearly four million inhabitants. Eligible patients were identified through medical records and included if they were ≥18 years of age and had a biopsy-confirmed diagnosis of (non-uveal) melanoma. Patients who started treatment between 2015-2023 were included. All patients were followed from the initiation of treatment until death, loss to follow-up, or the end of the study period (February 2025).

The following baseline variables were collected for all patients: age at treatment initiation, sex, ECOG performance status, lactate dehydrogenase (LDH) levels, melanoma subtype (cutaneous, acral, mucosal, or unknown primary), disease stage (classified as unresectable melanoma; III, M1a, M1b, M1c, or M1d according to the American Joint Committee on Cancer [AJCC] staging system, eight edition), previous therapies, and BRAF mutation status. The primary outcome was overall survival (OS), defined as the time from the start of treatment to death from any cause and patients alive at the end of the study were censored. Secondary outcomes included radiological response to treatment, progression-free survival (PFS) and irAEs. Objective response rate (ORR), included complete responses (CR) or partial response (PR), as defined according to the RECIST 1.1 criteria. Progression-free survival (PFS) was defined as the time from treatment initiation to documented disease progression or death, whichever occurred first. Immune-related AEs were classified and graded according to Common Terminology Criteria for Adverse Events (CTCAE) version 5.0. The study was approved by the Swedish Ethical Review Authority (Dnr 2023-04611-01 and Dnr 477-18).

*Statistical analysis*

Baseline characteristics between treatment groups (NIVO3+IPI1 versus NIVO1+IPI3) were compared using Student's t-test for age, Mann-Whitney U test for LDH levels, and Chi-square test for categorical variables (ECOG status, sex, disease stage, previous treatment, and BRAF status). Survival outcomes (OS and PFS) were analyzed using the Kaplan-Meier method. Cox proportional hazards regression models were used to estimate hazard ratios (HR) with 95% confidence intervals (CI) for OS and PFS, both unadjusted and adjusted for baseline covariates (age, ECOG, LDH level, disease stage and number of metastatic sites). Subgroup analyses were conducted to evaluate the treatment effect in the NIVO3+IPI1 and NIVO1+IPI3 groups across different patient populations, presented in forest plots for OS and PFS with adjustments in each subgroup for age, ECOG, LDH level, disease stage and number of metastatic sites). Adjusted hazard ratios (aHR) with 95% confidence intervals were calculated separately for the following predefined subgroups: sex (male or female), age (<60or ≥60 years), performance status (ECOG 0 or ≥1), BRAF mutation status (mutated or wild-type), disease stage (III+M1a+M1b or M1c or M1d), number of metastatic sites (<3 or ≥3), CNS metastasis burden (low or high, see **Supplementary Table S1**), melanoma type (cutaneous or mucosal), LDH level (<upper limit of normal (ULN or /≥ULN), and previous treatment with PD-1 and/or BRAF-MEK inhibitors (yes or no). The incidence of irAEs grade 3–5 was compared between groups using the Chi-square test, and the differences in radiological response was assessed using logistic regression. Given that the NIVO1+IPI3 regimen was implemented earlier than the NIVO3+IPI1 regimen in Swedish clinical practice, we conducted additional analyses to assess potential temporal bias. We compared the median start years between treatment groups using Student's t-test and performed sensitivity analyses by stratifying patients into early (≤June 2021) and late (≥July 2021) treatment cohorts. Cox regression analyses were performed separately for each temporal cohort to evaluate whether treatment effects varied over time. Stratified survival analyses were performed to further explore treatment effects within clinically relevant subgroups. Kaplan-Meier survival curves were generated for OS and PFS stratified by BRAF mutation status (mutated vs. wild-type) and disease stage (stage III+M1a+M1b, M1c, and M1d).

| **Table S1. Baseline characteristics of patients with advanced melanoma treated with NIVO3+IPI1 or NIVO1+IPI3** | | | |
| --- | --- | --- | --- |
| **Baseline characteristics** | **NIVO3+IPI1 (N=209)** | **NIVO1+IPI3 (N=190)** | **P-value** |
| **Age, median, years (IQR)** | 63 (55-74) | 60 (48-69) | <0.001 |
| **Sex, male** | 130 (62.2%) | 110 (57.9%) | 0.380 |
| **ECOG** |  | | 0.974 |
| *0* | 146 (69.9%) | 132 (69.5%) |  |
| *>0* | 63 (30.1%) | 58 (30.5%) |  |
| **BRAF mutated** | 98 (46.9%) | 105 (55.3%) | 0.095 |
| **Disease stage** |  | | <0.001 |
| *III* | 10 (4.8%) | 5 (2.6%) |  |
| *M1a* | 40 (19.1%) | 14 (7.4%) |  |
| *M1b* | 38 (18.2%) | 15 (7.9%) |  |
| *M1c* | 77 (36.8%) | 61 (32.1%) |  |
| *M1d* | 44 (21.1%) | 95 (50.0%) |  |
| **Site of metastases** |  |  |  |
| *Soft tissue* | 173 (82.8%) | 140 (73.7%) | 0.027 |
| *Lung* | 100 (47.8%) | 111 (58.0%) | 0.035 |
| *Liver* | 57 (27.3%) | 61 (32.1%) | 0.291 |
| *Bone* | 34 (16.3%) | 44 (23.2%) | 0.083 |
| *Other visceral* | 58 (27.8%) | 57 (30.0%) | 0.620 |
| *CNS* | 44 (21.1%) | 95 (50.0%) | <0.001 |
| **No. of metastatic sites, median (IQR)** | 2 (1-3) | 3 (2-4) | <0.001 |
| **No. of CNS metastases in M1d-patients** |  |  | 0.236 |
| *1* | 14 (31.8%) | 19 (20.0%) |  |
| *2* | 8 (18.2%) | 15 (25.8%) |  |
| *≥3* | 22 (50.0%) | 61 (64.2%) |  |
| **Largest CNS metastasis ≥1 cm** | 26 (60.5%) | 60 (69.8%) | 0.291 |
| **Corticosteroids due to CNS-metastases at NIVO+IPI initiation** | 10 (22.7%) | 26 (27.4%) | 0.561 |
| **All CNS metastases** **treated with radical surgery or SRS/SRT before NIVO+IPI** | 12 (27.3%) | 15 (15.8%) | 0.111 |
| **CNS metastases burden*** |  |  | 0.083 |
| **Low** | 18 (40.9%) | 25 (26.3%) |  |
| **High** | 26 (59.1%) | 70 (73.7%) |  |
| **Melanoma subtype** |  | | 0.614 |
| *Cutaneous melanoma* | 200 (95.7%) | 178 (93.7%) |  |
| *Mucosal melanoma* | 9 (4.3%) | 12 (6.3%) |  |
| **LDH > ULN** | 100 (47.9%) | 108 (56.8%) | 0.072 |
| **NIVO+IPI first line** | 91 (43.5%) | 85 (44.7%) | 0.810 |
| **NIVO+IPI second or third line** |  |  |  |
| *Previous treatment* |  | |  |
| *- BRAF (±MEK) inhibitors* | 21 (10.0%) | 45 (23.7%) |  |
| *- PD-1 inhibitor monotherapy* | 81 (38.8%) | 43 (22.6%) |  |
| *- BRAF-MEK inhibitors and PD-1 inhibitor monotherapy* | 13 (6.2%) | 14 (7.4%) |  |
| *- Other treatments* | 3 (1.4%) | 3 (1.6%) |  |
| **Year when NIVO+IPI was initiated, median (IQR)** | 2021 (2021-2022) | 2020 (2019-2022) | <0.001 |
| **Started NIVO+IPI June 2021 or earlier** | 82 (39.2%) | 111 (58.4%) |  |
| **Started NIVO+IPI July 2021 or later** | 127 (60.8%) | 79 (41.6%) |  |

*Low CNS metastases burden: Patients with all CNS metastases treated with surgery or stereotactic radiotherapy/stereotactic radiosurgery (SRT/SRS) before start of NIVO+IPI treatment OR those with maximum two CNS metastases, each less than 1 cm AND not symptomatic (patients not on cortisone). High CNS burden: All patients not falling into the criteria for low CNS burden.

ECOG = Eastern Cooperative Oncology Group performance status; LDH = lactate dehydrogenase; ULN = upper limit of normal.

| **Table S2. Best radiological response to treatment with NIVO3+IPI1 or NIVO1+IPI3 in patients with advanced melanoma.** | | | |
| --- | --- | --- | --- |
| **Radiological response** | **NIVO3+IPI1 (N=209)** | **NIVO1+IPI3 (N=190)** | **P-value** |
| **Best response, n (%)** | | | |
| *- CR* | 48 (23.0) | 29 (15.3) |  |
| *- PR* | 54 (25.8) | 29 (21.6) |  |
| *- SD* | 24 (11.5) | 17 (8.9) |  |
| *- PD* | 78 (37.3) | 98 (51.6) |  |
| *- Unknown* | 5 (2.4) | 5 (2.6) |  |
| **ORR (95% CI), %** | 48.8 (43.2-56.8) | 36.9 (31.1-45.1) | 0.016 |
| **DCR (95% CI), %** | 60.3 (54.9-68.2) | 45.8 (39.9-54.3) | 0.004 |

DCR includes CR, PR and SD. CR = complete response, PD = progressive disease, ORR = objective response rate; DCR = disease control rate; CI = confidence interval.

| **Table S3. Frequency and severity of irAEs in patients with advanced melanoma treated with NIVO3+IPI1 or NIVO1+IPI3** | | | | | | |  |  |
| --- | --- | --- | --- | --- | --- | --- | --- | --- |
|  | **NIVO3+IPI1 (N=209)** | | | **NIVO1+IPI3 (N=190)** | | |  | |
| **Type of irAE** | **Any grade** | **Grade 3-5** | | **Any grade** | | **Grade 3-5** |  | |
| **Fatigue** | 4 (1.5%) | 0 (0.0%) | | 2 (0.7%) | | 0 (0.0%) |  | |
| **Dermatological** | 60 (22.2%) | 1 (1.3%) | | 41 (15.0%) | | 2 (16.8%) |  | |
| **Endocrinological** | 57 (21.1%) | 9 (11.7%) | | 41 (15.0%) | | 12 (10.1%) |  | |
| **Gastrointestinal** | 86 (31.9%) | 47 (61.0%) | | 113 (41.4%) | | 70 (58.8%) |  | |
| **Pneumonitis** | 15 (5.6%) | 7 (9.1%) | | 19 (7.0%) | | 10 (8.4%) |  | |
| **Rheumatological** | 27 (10.0%) | 2 (2.6%) | | 33 (12.1%) | | 6 (5.0%) |  | |
| **Others** | 21 (7.8%) | 11 (14.3%) | | 24 (8.8%) | | 19 (16.0%) |  | |
| **Total** | 270 | | 77 | 273 | 119 | |  | |
|  |  | |  |  |  | | **P-value** | |
| **No. with grade 3-5 irAEs, % (n)** | 64 (30.6%) | | | 97 (51.1%)* | | | <0.001 | |
| **No. with irAEs treated**  **with corticosteroids, % (n)** | 126 (60.3%) | | | 121 (63.7%) | | | 0.699 | |
| **No. with irAEs leading**  **to discontinuation, % (n)** | 67 (32.1%) | | | 70 (36.8%) | | | 0.483 | |
| **No. with grade 3-5 irAEs leading**  **to discontinuation, % (n)** | 35 (16.7%) | | | 52 (27.4%) | | | 0.011 | |

Several types of AEs can be reported for the same patient.

*There was one grade 5 irAE, related to hematological toxicity.

irAE = immune-related adverse event.

| **Table S4. Post-progression treatment in patients with advanced melanoma treated with NIVO3+IPI1 or NIVO1+IPI3** | | | |
| --- | --- | --- | --- |
| **Later treatments** | **NIVO3+IPI1 (N=209)** | **NIVO1+IPI3 (N=190)** | **P-value** |
| **Post-progression treatment** | | | |
| *None* | 127 (60.8%) | 100 (52.6%) | 0.104 |
| *≥1 later treatment lines* | 82 (39.2%) | 90 (47.4%) |  |
| *- BRAF (±MEK) inhibitors* | 50 (23.9%) | 47 (24.7%) |  |
| *- PD-1 inhibitor rechallenge* | 10 (4.8%) | 9 (4.7%) |  |
| *- PD-1+CTLA-4 inhibitor rechallenge* | 7 (3.3%) | 10 (5.3%) |  |
| *- Chemotherapy* | 24 (11.5%) | 46 (24.2%) |  |

**
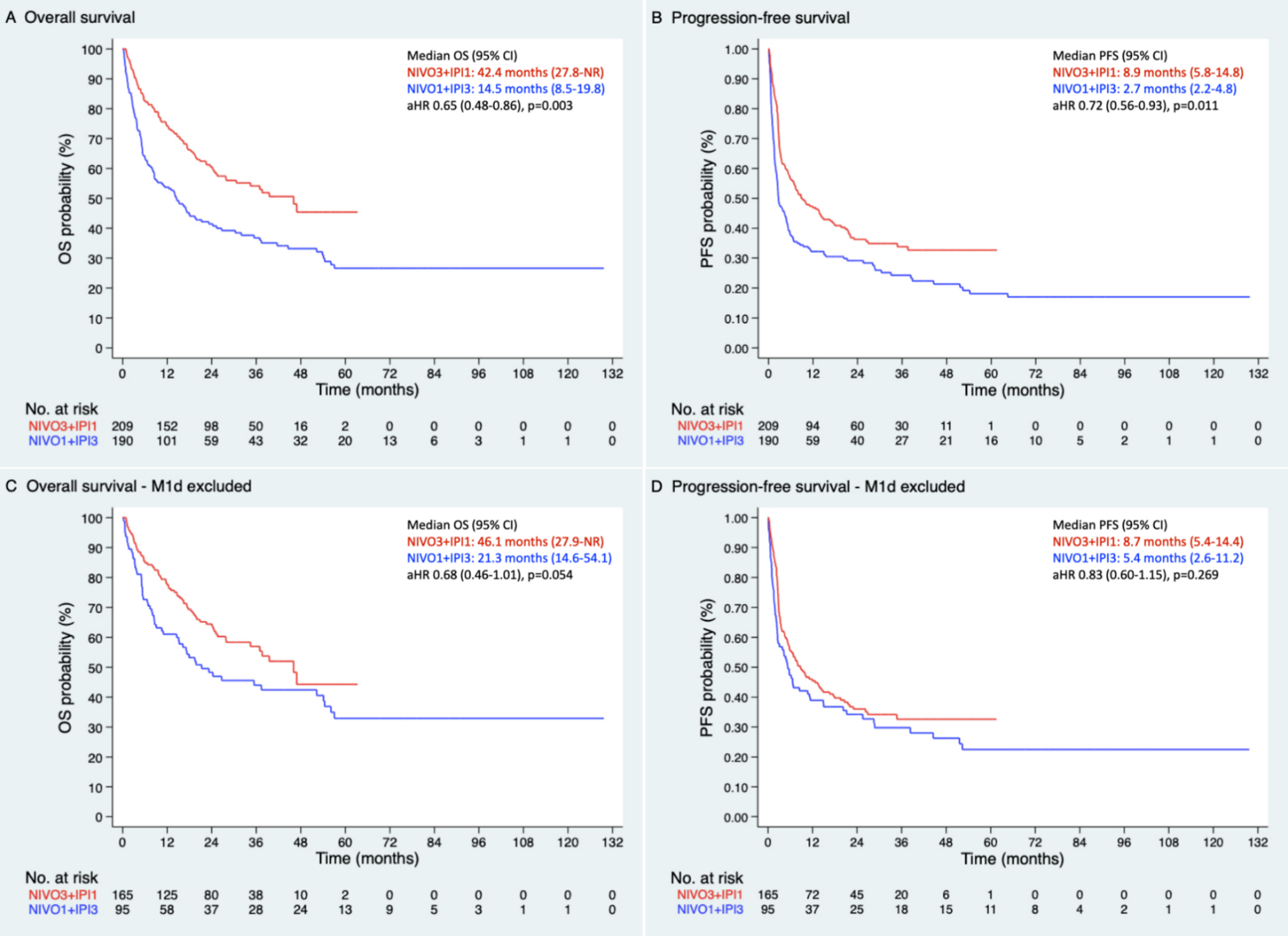
**

**Figure S1**. Kaplan-Meier curves for (A) OS and (B) PFS in patients with advanced melanoma treated with NIVO3+IPI1 or NIVO1+IPI3. OS = overall survival; PFS = progression-free survival; aHR = adjusted hazard ratio; CI = confidence interval; NR = not reached

**
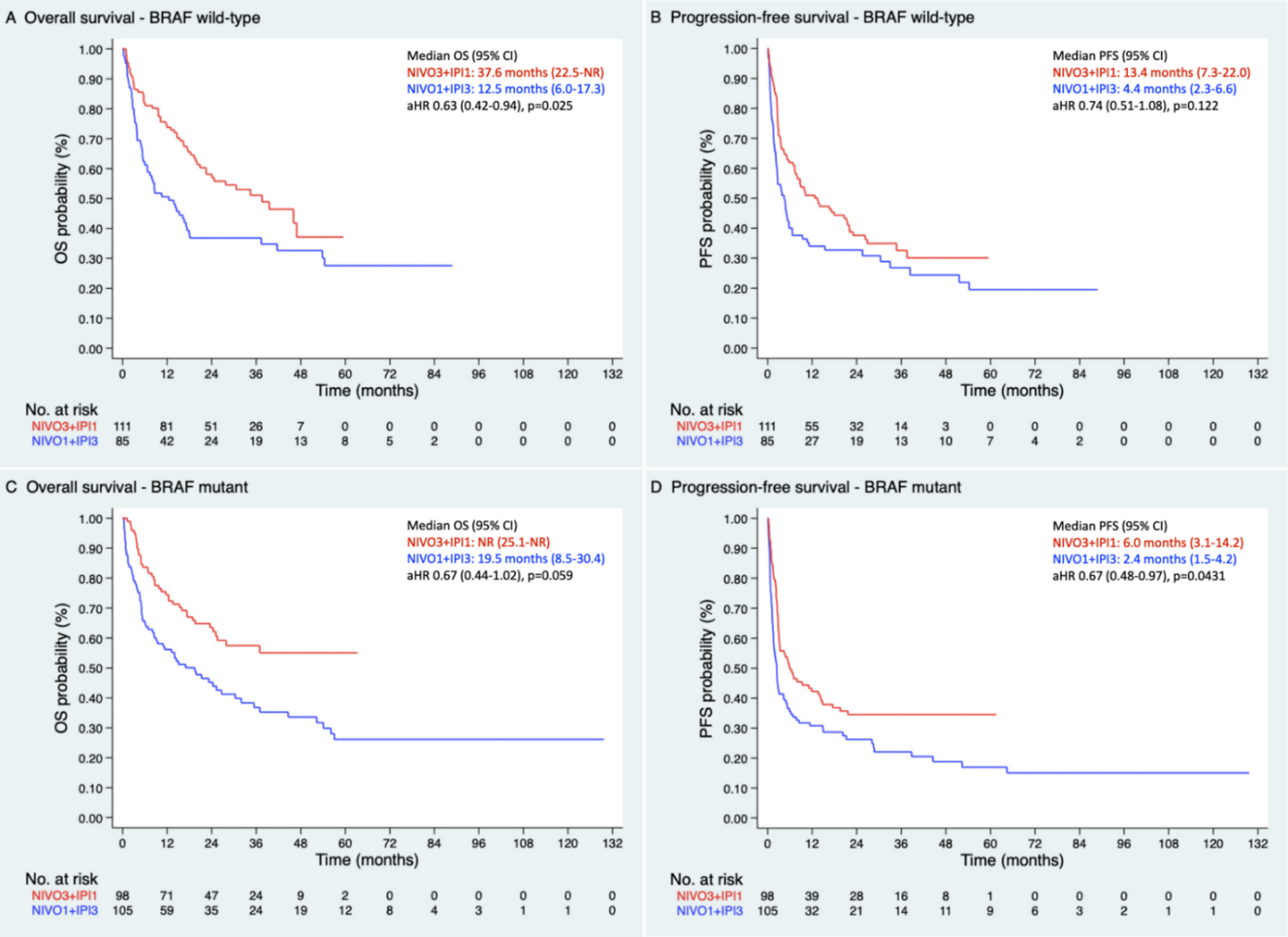
**

**Figure S2**. Kaplan-Meier curves for OS (A, C) and PFS (B, D) stratified by BRAF mutation status. OS = overall survival; PFS = progression-free survival; aHR = adjusted hazard ratio; CI = confidence interval; NR = not reached.

**
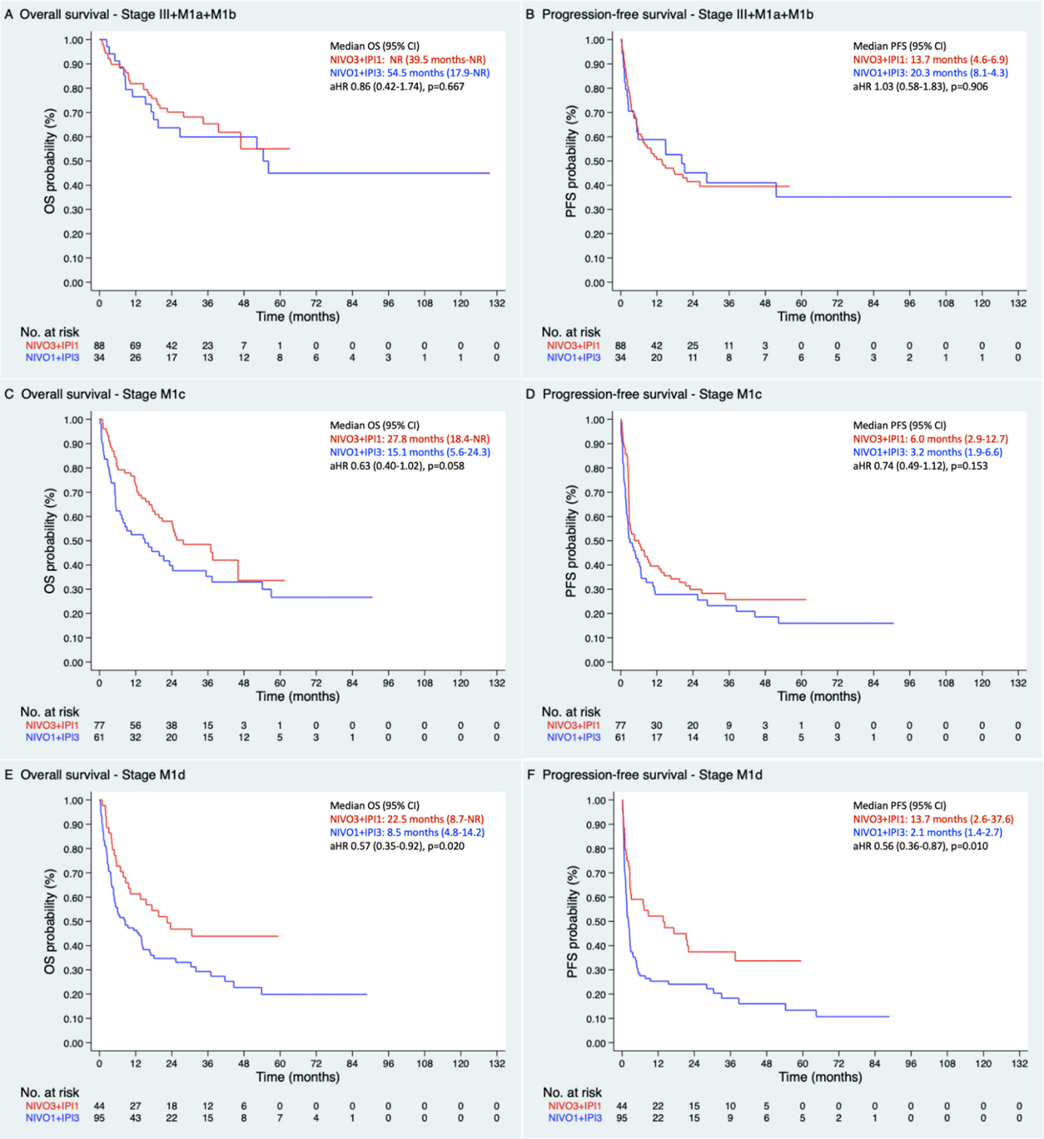
**

**Figure S3.** Kaplan-Meier curves for OS (A, C, E) and PFS (B, D, F) stratified by disease stage. OS = overall survival; PFS = progression-free survival; aHR = adjusted hazard ratio; CI = confidence interval; NR = not reached.
